# Role Of MMP-2 and MMP-9 in Resistance to Drug Therapy in Patients with Resistant Hypertension

**DOI:** 10.5935/abc.20150060

**Published:** 2015-08

**Authors:** Leandro Lacerda, Ana Paula de Faria, Vanessa Fontana, Heitor Moreno, Valéria Sandrim

**Affiliations:** 1 Núcleo de Pós-Graduação e Pesquisa - Santa Casa de Belo Horizonte, Belo Horizonte, MG – Brazil; 2 Faculdade de Ciências Médicas da UNICAMP, Campinas, SP – Brazil

**Keywords:** Matrix Metalllooproteinases, Hypertension/physiopathology, Endopeptidases, Hyperaldosteronism/physiopathology

## Abstract

**Background:**

Despite the increased evidence of the important role of matrix metalloproteinases (MMP-9 and MMP‑2) in the pathophysiology of hypertension, the profile of these molecules in resistant hypertension (RHTN) remains unknown.

**Objectives:**

To compare the plasma levels of MMP-9 and MMP-2 and of their tissue inhibitors (TIMP-1 and TIMP-2, respectively), as well as their MMP-9/TIMP-1 and MMP-2/TIMP-2 ratios, between patients with controlled RHTN (CRHTN, n=41) and uncontrolled RHTN (UCRHTN, n=35). In addition, the association of those parameters with clinical characteristics, office blood pressure (BP) and arterial stiffness (determined by pulse wave velocity) was evaluate in those subgroups.

**Methods:**

This study included 76 individuals diagnosed with RHTN and submitted to physical examination, electrocardiogram, and laboratory tests to assess biochemical parameters.

**Results:**

Similar values of MMP-9, MMP-2, TIMP-1, TIMP-2, and MMP-9/TIMP-1 and MMP-2/TIMP-2 ratios were found in the UCRHTN and CRHTN subgroups (P>0.05). A significant correlation was found between diastolic BP (DBP) and MMP-9/TIMP-1 ratio (r=0.37; P=0.02) and DPB and MMP-2 (r=-0.40; P=0.02) in the UCRHTN subgroup. On the other hand, no correlation was observed in the CRHTN subgroup. Logistic regression models demonstrated that MMP-9, MMP-2, TIMP-1, TIMP-2 and their ratios were not associated with the lack of BP control.

**Conclusion:**

These findings suggest that neither MMP-2 nor MMP-9 affect BP control in RHTN subjects.

## Introduction

Resistant hypertension (RHTN) is a clinical condition characterized by maintenance of blood pressure (BP) levels above goal (140/90 mm Hg), despite the concurrent use of three or more antihypertensive agents of different classes. Ideally, one of these drugs should be a diuretic, and all agents should be prescribed at optimal doses [subgroup called uncontrolled RHTN (UCRHTN)]. The subgroup of resistant hypertensive patients whose BP is controlled using four or more drugs is known as controlled RHTN (CRHTN)^1^.

Matrix metalloproteinases (MMPs), a group of zinc- and calcium-dependent endopeptidases, and their endogenous tissue inhibitors (TIMPs) are primarily responsible for stromal matrix remodeling^2^. Currently, some evidence has also suggested that those molecules play a role in hypertensive processes^3^.

Experimental hypertension studies have reported that the intima and media thickness of conduct vessels was associated with increased expression of MMP-9 and MMP-2, and this event could be prevented with non-selective MMP inhibitor (doxycycline) treatment^4,5^. Previous studies have found that MMP-2 is upregulated in response to high intra-luminal pressure^6^, and its increased levels have been reported in the mammary arteries of hypertensive subjects^7^. Evidence has suggested that MMP-2 can degrade big endothelin-1, thus promoting vasoconstrictor effect^8^. Matrix metalloproteinases have been shown to suppress the vasodilation induced by β-agonists in hypertensive rats^9^. In hypertensive patients, increased MMP-9 activity may lead to degradation of elastin, while reduced TIMP-1 activity can lead to accumulation of fibrin degradation products, resulting in misdirected deposition of collagen^10^.

These experimental studies have stimulated further investigation of MMPs and TIMPs as potential biomarkers in hypertension. The circulating concentration of these molecules may be associated with hypertension complications and prognosis, being therefore useful in clinical practice^11^. In addition, MMP-2, MMP-9, TIMP-1 and TIMP-2 may be directly associated with RHTN, playing a role in BP control in those patients^12^.

Although plasma MMP-9, MMP-2, TIMP-1 and TIMP-2 levels have been measured in hypertensive subjects^13^, these concentrations in RHTN patients are unknown. The present study is the first to compare the plasma levels of those molecules, as well as their ratios (MMP-9/TIMP-1 and MMP-2/TIMP-2) between CRHTN and UCRHTN patients.

## Methods

### Patient population

This cross-sectional study included all 76 individuals diagnosed with RHTN on regular follow-up at the Resistant Hypertension Outpatient Clinic, University of Campinas, Campinas, Brazil. Patients were classified into two subgroups, UCRHTN (n=35) and CRHTN (n=41), in accordance with the guidelines established by the American Heart Association^14^.

All patients underwent physical examination, electrocardiogram, and laboratory tests to assess biochemical parameters. Patients with secondary forms of hypertension as well as renal failure, ischemic heart, liver and peripheral vascular diseases, stroke, smoking or any other serious disease were properly identified and excluded from the study. Ambulatory BP monitoring was performed (Spacelabs 90207, Spacelabs Inc, Redmond, WA, USA) to exclude pseudo-resistant hypertension and to characterize CRHTN and UCRHTN patients. Treatment adherence was determined by pill counting (threshold of 80% or greater of the prescribed medication).

This study was approved by the Research Ethics Committee at the Medical Sciences School, University of Campinas, Campinas, Brazil, and was performed in accordance with the Declaration of Helsinki. All participants were aware of the nature of the research study and signed an informed consent before enrolling in the study.

The following patients’ parameters were evaluated: office BP; pulse wave velocity (PWV); plasma concentrations of MMP-9, MMP-2, TIMP-1 and TIMP-2; plasma aldosterone concentration (PAC); and plasma renin activity (PRA).

### Office BP measurements

Systolic and diastolic BP (SBP and DBP, respectively) levels were assessed three times, using a digital sphygmomanometer (Omron HEM-711DLX, OMRON Healthcare Inc., Bannockburn, IL, USA) on the right upper arm, in the sitting position, after a 10-minute rest. The mean of two consecutive measurements was used, with a variation lower than 5 mmHg.

### Pulse wave velocity assessment

Pulse wave velocity was measured by using the Sphygmocor System (Atcor Medical, Sydney, Australia) with the patient in the supine position^15^. The PWVs of the right carotid and femoral arteries were analyzed, estimating the delay with respect to the electrocardiogram wave. Distance measurements were taken between the femoral recording site and the supra-sternal notch minus the distance from the supra-sternal notch to the carotid recording site. Carotid-femoral PWV was calculated by dividing the traveled distance by transit time [PWV = distance(m)/time(s)]. At least two measurements were performed; if they differed by more than 0.5 m/s, a third measurement was taken.

### Laboratory assessments

Blood samples for biochemical assessment were collected at 8 AM, after an overnight fasting. PAC and PRA were measured by using radioimmunoassay, with standard techniques. Plasma levels of biomarkers MMP-9 and TIMP-1 were measured by using enzyme-linked immunosorbent assay (ELISA) (R&D System®, Minneapolis, USA). Similarly, the plasma biomarkers MMP-2 and TIMP-2 were measured by using ELISA, following the manufacturer´s instructions (RayBiotech®, Georgia, USA).

### Statistical analyses

The Statistical Analysis System, version 3.02 (GraphPad Prism Inc., 2000), and SigmaPlot version 12.0 (Systat software, Inc.) were used for all statistical analyses of the study.

All values were expressed as mean ± standard deviation. The normality of distribution was assessed by using Kolmogorov–Smirnov test. The subgroups were compared by using Student´s *t* test or Mann-Whitney test, according to data distribution. Chi-square test was used for categorical variables. The correlation of biomarkers with clinical parameters was evaluated by using Pearson’s or Spearman´s test. Regression models were performed to test the association of variables apart from potential confounders. The level of significance accepted was 0.05.

## Results

Table 1 shows the clinical and laboratory data of both subgroups, and Table 2 shows the plasma levels of biomarkers. As expected, increased values of SBP, DBP and PWV were found in UCRHTN as compared to CRHTN patients. No significant differences were observed regarding age, sex, body mass index (BMI) and biochemical parameters. Similar values of MMP-9, TIMP-1, MMP-2, TIMP-2, and of MMP-9/TIMP-1 and MMP-2/TIMP-2 ratios were found in the UCRHTN and CRHTN subgroups (P>0.05; Table 2).

**Table 1:** General characteristics of the resistant hypertension (RHTN) subgroups

	**UCRHTN (n= 35)**	**CRHTN (n= 41)**
Female gender (%)	63	66
Age (years)*	57 ± 11	61 ± 9
BMI (Kg/m2)	30.0 ± 4.4	30.1 ± 4.4
SBP (mm Hg)*	158 ± 20	136 ± 14
DBP (mm Hg)*	91 ± 14	80 ± 7
PWV (m/s) *	11.9 ± 1.8	10.6 ± 1.3
Total cholesterol (mg/dL)	203 ± 50	202 ± 39
LDL (mg/dl)	126 ± 38	125 ± 35
HDL (mg/dl)	44 ± 12	48 ± 14
Triglycerides (mg/dL)	160 ± 96	149 ± 66
Urea (mg/dL)	38.1 ± 11.8	35.9 ± 7.5
Creatinine (mg/dL)	1.0 ± 0.2	0.9 ± 0.2
Fasting glucose (mg/dL)	125.4 ± 54.1	106.7 ± 34.2
Uric acid (mg/dL)	5.9 ± 1.6	5.8 ± 1.5
Aldosterone (pg/mL) *	109.7 ± 82.0	101.1 ± 70.5
Renin (pg/mL)	22.4 ± 19.6	21.2 ± 18.2
Antihypertensive drugs Total number (daily) *	**4.6 ± 0.9**	**4.2 ± 0.9**
Spironolactone (%)	43	37
Diuretics (%)	100	100
Beta-blockers (%)	69	68
ACEI (%)	46	29
ARB (%)	54	51
CCB (%)*	97	68
Centrally acting anti-hypertensive (%)	37	17

UCRHTN: uncontrolled resistant hypertension; CRHTN: controlled resistant hypertension; BMI: body mass index; SBP: systolic blood pressure; DBP: diastolic blood pressure; PWV: Pulse wave velocity; LDL low density lipoprotein; HDL: High density lipoprotein; ACEI: Angiotensin-converting-enzyme inhibitors; ARB; Angiotensin II receptor blockers; CCB: Calcium channel blockers. Values are expressed as mean ± SD or percentage.

* p <0.05 between groups.

**Table 2:** Characteristics of biomarkers in resistant hypertension (RHTN) subgroups

**Biomarkers**	**UCRHTN (N= 35)**	**CRHTN (N= 41)**
MMP-9 (ng/mL)	253 ± 134	225 ± 121
TIMP-1 (ng/mL)	499 ± 406	407 ± 249
MMP-9/TIMP-1 Ratio	0.68 ± 0.45	0.77 ± 0.59
MMP-2 (ng/mL)	330 ± 71	312 ± 69
TIMP-2 (ng/mL)	306 ± 132	339 ± 184
MMP-2/TIMP-2 Ratio	1.31 ± 0.79	1.24 ± 0.78

UCRHTN: uncontrolled resistant hypertension; CRHTN: controlled resistant hypertension; MMP-9: Matrix metalloproteinase-9; TIMP-1: tissue inhibitor MMP-1; MMP-2: Matrix metalloproteinase-2; TIMP-2: tissue inhibitor MMP-2. Values are expressed as mean ± SD or percentage. * p <0.05 between groups.

Regarding antihypertensive medication, UCRHTN patients were taking a significantly higher number of anti-hypertensive drugs, demonstrated by the use of calcium channel blockers, as compared to controlled subjects (Table 1).

Correlation analyses for the UCRHTN subgroup indicated that DBP correlated with MMP-9/TIMP-1 ratio (r=0.37; P=0.02); however, DBP was inversely correlated with MMP-2 levels (r=-0.40; P=0.02). In that subgroup, PAC and age also correlated with MMP-9/TIMP-1 ratio (r = 0.57, p <0.001 and r = -0.37, P = 0.02, respectively), and, only in that subgroup, MMP-2 correlated with age (r = 0.42, p = 0.01). In addition, these associations remained significant after adjusting for sex and BMI included in the linear regression model [beta coefficient=11.5, standard error (SE)=5.5, p=0.04; beta coefficient=-0.08, SE=0.04, p=0.04, respectively]. Finally, the plasma levels of the biomarkers mentioned above did not correlate with any clinical parameter in CRHTN subjects (Table 3 and 4). Considering the entire RHTN group (n=76), we found that (i) the MMP-9/TIMP-1 ratio was inversely associated with BMI (r=-0.25, p=0.03), but positively with aldosterone levels (r=0.24, p=0.04); and (ii) MMP-2 was inversely associated with DBP (r=-0.26, p=0.02), but positively with age (r=0.40, p<0.001). Finally, logistic regression models demonstrated that MMP-9 and MMP-2, their tissue inhibitors-1 and -2 and ratios were not associated with the lack of BP control (data not shown) in RHTN when adjusting for sex, age and BMI.

**Table 3:** Correlation among clinical parameters and *MMP-9, TIMP-1* and *MMP-9/TIMP-1*

**Groups**	**Biomarkers**	**SBP**	**DBP**	**PWV**
	*MMP-9*	0.23 (0.14)	0.06 (0.67)	0.06 (0.66)
CRHTN	*TIMP-1*	0.03 (0.85)	0.12 (0.44)	-0.06 (0.67)
r(p-value)	*MMP-9/TIMP-1 Ratio*	0.07 (0.64)	-0.12 (0.42)	-0.02 (0.86)
	*MMP-9*	0.04 (0.82)	0.14 (0.41)	-0.03 (0.82)
UCRHTN	*TIMP-1*	-0.23 (0.17)	-0.33 (0.05)	-0.07 (0.68)
r (p-value)	*MMP-9/TIMP-1 Ratio*	0.15 (0.38)	0.37 (0.02*)	0.02(0.91)

Data are expressed as correlation coefficient (p-value). CRHTN: Controlled resistant hypertension; UCRHTN: Uncontrolled resistant hypertension; SBP: systolic blood pressure; DBP: Diastolic blood pressure, PWV: Pulse wave velocity.

* p <0.05.

**Table 4:** Correlation among clinical parameters and MMP-2, TIMP-2 and MMP-2/TIMP-2

**Groups**	**Biomarkers**	**SBP**	**DBP**	**PWV**
	*MMP-2*	-0.01 (0.93)	0.02 (0.88)	-0.09 (0.54)
CRHTN	*TIMP-2*	-0.14 0.25)	-0.03 (0.83)	0.04 (0.77)
r (p-value)	*MMP-2/TIMP-2 Ratio*	0.23 (0.13)	0.04 (0.75)	-0.24 (0.12)
	*MMP-2*	-0.21 (0.20)	-0.40 (0.02*)	0.18 (0.29)
UCRHTN	*TIMP-2*	0.21 (0.21)	-0.01 (0.97)	0.03 (0.85)
r (p-value)	*MMP-2/TIMP-2 Ratio*	-0.26 (0.11)	-0.28 (0.09)	0.06 (0.72)

Data are expressed as correlation coefficient (p-value). CRHTN: Controlled resistant hypertension; UCRHTN: Uncontrolled resistant hypertension; SBP: Systolic blood pressure; DBP: Diastolic blood pressure, PWV: Pulse wave velocity.

* p <0.05.

## Discussion

This is the first study to analyze the association of the biomarkers MMP-2 and MMP-9 with BP levels in the RHTN population. Interestingly, correlations of DBP and age with the MMP-9/TIMP-1 ratio and DBP and MMP-2 were observed only in the UCRHTN subgroup. Plasma aldosterone levels and age also correlated with the MMP-9/TIMP-1 ratio in UCRHTN. In this context, as previously demonstrated^1,16,17^, the idea of several important differences in the pathophysiology of the RHTN subgroups should be reinforced. However, no association of the biomarkers with SBP was found, probably because DBP is a more stable variable than the systolic component.

Under physiological conditions, balance between MMPs and TIMPs exists. On the other hand, in pathological processes, such as hypertension, an MMPs/TIMPs ratio imbalance contributes to the excessive degradation of extracellular matrix (ECM) proteins^18^, and results in pathological vascular remodeling^19^ . Therefore, the MMP-9/TIMP-1 ratio might be a better indicator of that process. Taken together, MMP-9/TIMP-1 ratio in association with DBP levels in UCRHTN could strengthen the importance of some different phenotypes in the pathophysiology of uncontrolled patients.

Inconsistent results have been found about the levels of gelatinases (MMP-2 and MMP-9) in essential hypertension^3^. However, our study differs from this previous finding in evaluating gelatinases and their inhibitors in RHTN. It is well known that RHTN is associated with increased cardiovascular risk^20^, but uncontrolled hypertensive patients are probably exposed to increased cardiovascular risk, which may reflect in a worse prognosis as compared to controlled subjects. Moreover, our study found an inverse correlation between MMP-2 and DBP in the UCRHTN subgroup, suggesting no association between MMP-2 and BP control in that subgroup.

Matrix metalloproteinases are zinc-dependent endopeptidases, with that ion in the active site. Likewise, the angiotensin-converting-enzyme (ACE) is also zinc-dependent and inhibited by ACE inhibitors, which are widely used in current antihypertensive treatment. Given this, MMP-9 may also be inhibited by ACE inhibitors by binding with zinc in the active site^21^; this suggests that treatment with ACE inhibitors may inhibit MMP-9 activity^22^.

Although high MMP-9 levels were expected in the UCRHTN subgroup, this negative finding may be explained by the fact that all RHTN individuals have the hypertensive disease for a long time and take a great number of antihypertensive drugs, which could cause the decrease in MMP-9 activity, particularly related to the use of ACE inhibitors, as evidenced by several studies^21,22^.

For example, some studies have evaluated the relationship between MMP-9 and TIMP-1 in patients with essential hypertension, and have shown that, after antihypertensive treatment, the circulating levels of those molecules were significantly higher in subjects with hypertension than in normotensive controls. In some cases, a reduction in plasma levels of MMP-9 and consequent increased levels of TIMP-1 have occurred after antihypertensive treatment^23^. Other findings are as follows: MMP changes in TIMP profile, which favor decreased ECM degradation (decreased MMP-2, MMP-9 and MMP-13 and increased TIMP-1), are associated with left ventricular hypertrophy and diastolic dysfunction; and increased TIMP-1 predicted the presence of chronic heart failure^11^.

In addition, significantly higher TIMP-1 levels have been reported in hypertensive individuals as compared with normotensive individuals; however, TIMP-1 levels are not elevated in hypertension alone, but only in patients with diastolic dysfunction and fibrosis. This suggests that TIMP-1 synthesis and release are independent of BP and probably dependent on a variety of neurohormonal factors, being a TIMP-1 level higher than 500 ng/mL an accurate indicator of dysfunction diastolic and damage to target organs^24^.

One hypothesis to be raised about the increase of plasma levels of TIMP-1 is to generate a response to modulate or limit collagen degradation, thus contributing to the development of arterial stiffness. Unlike MMP-9, some studies indicate an increase of TIMP-1 after antihypertensive treatment^10,23,24^.

In contrast, some studies have reported that increased TIMP-1 levels were associated with an increased incidence of hypertension and risk of BP progression^25^. Other studies have shown the increase of TIMP-1 in normotensive *vs*. hypertensive subjects^26^, as well as unchanged^27^ or decreased TIMP-1^28^.

In addition, TIMPs play an important role in cardiovascular remodeling processes, regardless of their MMP inhibitory activity, ie, such inhibitors may play an important role in BP, irrespective of the action of MMPs^23^.

Pulse wave velocity is widely used as an arterial elasticity and stiffness index, and the arterial wall properties, such as thickness and lumen diameter, are the factors that most influence PWV^29^. Pulse wave velocity is the gold standard method to measure arterial stiffness, plays an essential role in the pathophysiology of hypertension and predicts mortality in patients with hypertension^30^. The mechanisms involved in arterial stiffness are not completely understood; however, evidence has shown that this process is accompanied by complex mechanisms, including structural alterations of the ECM, including the participation of MMPs. In our study, the levels of gelatinases and TIMPs were not correlated with PWV values. These negative findings may be related to vascular stiffness in RHTN, as previously shown^31^. In addition, the stiffness of great arteries appears to be an inevitable consequence of aging, ie, this process becomes more pronounced at older ages, which, according to the authors, is the most important determinant of arterial stiffness^1^. In this study, the arterial stiffness process may have been completed or lost, because the individuals were in advanced age, which is directly related to the increase in PWV and pulse pressure (PP), especially in the UCRHTN group. This may be an explanation for the lack of correlation of the biomarkers studied with PWV and PP.

Primary aldosteronism is the second most common cause of RHTN^32^. This condition is characterized by excessive secretion of aldosterone by the adrenal gland, the major forms being the production of adenomas and idiopathic hyperaldosteronism^33,34^.

It is noteworthy that patients with RHTN have increased aldosterone levels, but that is not due to primary aldosteronism. Previous works have shown that UCRHTN individuals have higher PAC as compared to CRHTN individuals^1^. A study of 88 consecutive patients with RHTN has reported a 20% incidence of primary aldosteronism, defined by measuring two parameters: PRA and urinary aldosterone concentration^35^. Consistent with these findings, other medical centers have reported a 17%–22% prevalence of primary aldosteronism in RHTN patients^36,37^. High PAC leads to the remodeling of small and large arteries, causing collagen synthesis, which results in increased arterial stiffness and BP elevation^38^.

Although we found a positive correlation between PAC and MMP-9/TIMP-1 ratio, hyperaldosteronism is known to be an independent risk factor in arterial hypertension and, thus, in the process of arterial stiffening^32^.

The main limitation of this study was the small number of UCRHTN and CRHTN patients enrolled. This study’s sample size was not calculated, because all 76 subjects on regular follow-up at the Resistant Hypertension Outpatient Clinic were included. Similarly, recent studies have demonstrated important findings, including in CRHTN and UCRHTN, with such a small population^16,17,39^. On the other hand, the lack of association in the main findings may be attributed to low statistical power or type II error. Moreover, antihypertensive drugs can influence the levels of MMP-9, as demonstrated by Fontana et al.^13^ and other studies previously cited. Multiple linear regression analyses was performed to predict biomarkers (MMP-2, MMP-9, TIMP-1, TIMP-2, and their ratios) adjusted for antihypertensive drugs. These regression models indicated that only the beta-blocker use was a predictor of TIMP-1 levels and of MMP-9/TIMP-1 ratio in all RHTN subjects. However, this potential confounding factor did not affect our findings, because both controlled and uncontrolled subgroups had a similar proportion of beta-blocker use. Because of ethical concerns, antihypertensive drugs could not be withdrawn in the RHTN subjects to exclude the influence of those medications on the plasma levels of biomarkers.

## Conclusion

Briefly, although MMP-9/TIMP-1 ratio and MMP-2 were associated with DBP levels, aldosterone and age in the UCRHTN subgroup, this does not seem to influence resistance to antihypertensive therapy, because the biomarkers did not predict the lack of BP control in RHTN. Future prospective studies with a larger RHTN population should be carried out to confirm the present study’s findings.
